# Concurrent gastro-intestinal nematode infection does not alter the development of experimental cerebral malaria

**DOI:** 10.1016/j.micinf.2008.04.015

**Published:** 2008-07

**Authors:** Brian de Souza, Helena Helmby

**Affiliations:** Immunology Unit, Department of Infectious and Tropical Diseases, London School of Hygiene and Tropical Medicine, Keppel Street, London WC1E 7HT, UK

**Keywords:** Malaria, Helminth, Pathology

## Abstract

Concurrent helminth infections have been suggested to be associated with protection against cerebral malaria in humans, a condition characterised by systemic inflammation. Here we show that a concurrent chronic gastro-intestinal nematode infection does not alter the course of murine cerebral malaria. Mice infected with *Heligmosomoides polygyrus*, and co-infected with *Plasmodium berghei* ANKA 14 days later, developed malaria parasitemia, weight loss and anemia, at the same rate as mice without nematode infection. Both groups developed cerebral malaria around the same time point. The data suggest that a chronic helminth infection does not affect the development of cerebral malaria in a murine model.

## 1 Introduction

Cerebral malaria (CM) is a major cause of death in humans infected with *Plasmodium falciparum* malaria, mainly in children in sub-Saharan Africa. CM is characterised by impaired consciousness and convulsions, with sequestration of parasitized erythrocytes in the microvasculature of the brain and the release of pro-inflammatory cytokines and other immune mediators contributing to the neurological symptoms [1]. Infections with the rodent malaria *Plasmodium berghei* ANKA (PbA) result in experimental cerebral malaria in susceptible strains of mice, such as C57BL/6. The CM caused by PbA infection displays many features of human CM, and has been used extensively to identify factors involved in CM pathogenesis (reviewed in Ref. [2]).

CD4^+^ CD25^+^ regulatory T cells (Treg) are naturally occurring regulatory T cells that express the transcription factor FoxP3. These cells play a critical role in maintaining self-tolerance and control of immune reactivity. Tregs can mediate their suppressive effects via cell contact, or via secretion of soluble factors such as the anti-inflammatory cytokines IL-10 and/or TGF-β. Regulatory T cells are rapidly induced in humans infected with *P. falciparum* and associated with higher rates of parasite growth [3]. Furthermore, depletion of Tregs prevent the development of CM in susceptible mice, and this is associated with improved CD4 and CD8 T cell activation and reduced accumulation of parasitized erythrocytes in the brain vasculature [4], again suggesting a damaging role for Tregs in malaria pathology.

Helminth infections cause some of the most prevalent and chronic human diseases worldwide [5] and are often associated with immune down-regulation and modulation [6]. These regulatory responses are known to play an important part in balancing the immune response against the different life stages of these parasites [6]. The murine gastro-intestinal nematode *Heligmosomoides polygyrus* have been extensively used to model chronic helminth infections and several studies have demonstrated the development and functional activity of *H. polygyrus*-induced CD4^+^ CD25^+^ T regulatory cells [7–9].

The possibility of interactions between helminth and malaria infections has been the subject of great interest in recent years (reviewed in Ref. [10] and [11]). In order to investigate whether helminth-induced immune modulation alters the course of murine CM we infected mice first with *H. polygyrus* and then with *P. berghei* ANKA and monitored the development of cerebral pathology. Our findings demonstrate that *H. polygyrus* infection does not alter the development of murine CM.

## 2 Material and methods

### 2.1 Animals and infections

Six- to eight-week-old C57BL/6 and BALB/c mice were bred at the animal unit, LSHTM under SPF conditions. All experiments were performed under the regulations of the Home Office Scientific Procedures Act (1986). Experimental animals (10–15 per group) were infected with 200 *H. polygyrus* L3 larvae by oral gavage. Fourteen days later groups of *H. polygyrus* infected or uninfected mice were infected with 10^4^ asexual bloodstage *P. berghei* ANKA malaria parasites i.v. Maintenance and infection of *P. berghei* ANKA were as described previously [12]. *H. polygyrus* was originally obtained from Prof. J. M. Behnke, Nottingham and maintained as stock infection in CD-1 mice. Infective L3 larvae were cultured as described by Bryant [13]. Control groups receiving single infections were infected in parallel for each experiment. Malaria parasitemia was determined on Giemsa stained thin blood smears and the level of hemoglobin in peripheral blood was analysed using a HemoCue hemoglobin analyser (HemoCue, Dronfield, UK). Animals were monitored daily for clinical signs of cerebral malaria (head deviation, convulsions, ataxia and paraplegia), and euthanised when appropriate to avoid unnecessary suffering according to Home Office regulations. Blood–brain barrier function was analysed by Evans blue assay. Animals showing clinical signs of cerebral symptoms, and controls, were injected with 200 μl 2% Evans blue (Sigma, Gillingham, UK) i.p. Sixty minutes later animals were terminally anesthetised, bled and perfused using 10 ml of cold saline. The perfused brains were removed, weighed and placed in 1 ml of dimethylformamide for 24 h. The amount of Evans blue in brain and plasma was analysed by spectrophotometry at 620 nm, using a standard curve of known concentration of Evans blue. Values are expressed as microgram Evans blue per gram brain tissue, normalised to plasma levels (microgram per ml) of each individual animal (Evans blue units/gram tissue).

### 2.2 Cytokine analyses

Serum cytokine analyses were carried out using routine sandwich ELISAs for IFN-γ (Mabtech AB, Nacka, Sweden), TNF-α and IL-10 (R&D systems, Abingdon, UK).

### 2.3 Statistical analyses

Significant differences (*p* < 0.05) between experimental groups were determined using the logrank test for survival and student’s *t*-test for all other analyses.

## 3 Results

### 3.1 Concurrent *H. polygyrus* infection does not alter the development of cerebral malaria

**Fig. 1:**
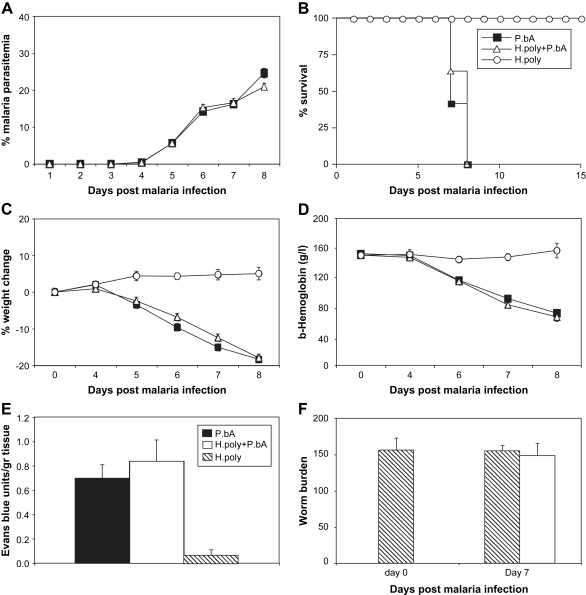
Concurrent *H. polygyrus* infection does not alter the development of cerebral malaria. Groups of C57BL/6 mice were inoculated orally with 200 *H. polygyrus* larvae. Fourteen days later they were co-infected with 10^4^*P. berghei* ANKA infected red blood cells i.v. (white triangles). Control mice infected with malaria only (black squares) or *H. polygyrus* only (white circles) were analysed in parallel. The development of malaria parasitemia (A), mortality (B), changes in body weight (C), hemoglobin levels (D), blood–brain barrier function by Evans blue assay on day 7 (E), and *H. polygyrus* worm burdens before and after malaria infection (F), were assessed. Mean and SEM are shown. Data is pooled data from two separate experiments involving a total of 25 animals per group.

Groups of C57BL/6 mice were infected with 200 *H. polygyrus* larvae by oral gavage. Fourteen days later establishment of the helminth infection was confirmed by worm counts (Fig. 1F). Around this time point strong helminth-induced Th2 and T regulatory responses can be detected ([7] and our unpublished observations), and we, therefore, infected mice with and without *H. polygyrus* infection with 10^4^ bloodstage PbA i.v. Control groups were infected with only PbA or only *H. polygyrus*. Parasitemia, weight loss and anemia were followed in all the individual mice. The data in Fig. 1 show that mice harbouring a *H. polygyrus* infection were no more, or less, susceptible to PbA infection than non-helminth-infected mice. Both groups developed comparable levels of malaria parasitemia, weight loss and anemia (Fig. 1A, C and D) and started to develop CM symptoms (head deviation, convulsions, ataxia and paraplegia) by day 7 post PbA infection, and all mice succumbed to CM by day 8. There was no statistical difference in the time to first symptoms or death between the two groups (Fig. 1B). Blood–brain barrier function was analysed by Evans blue assay at the time of first symptoms of CM. As can be seen in Fig. 1E the level of vascular leakage in the brain was similar between single and co-infected mice. As expected, mice infected with only *H. polygyrus* had minimal vascular leakage. Similar levels of worm burdens in the groups were confirmed before and after malaria infection (Fig. 1F).

### 3.2 Helminth co-infection does not alter systemic cytokine responses

**Fig. 2:**
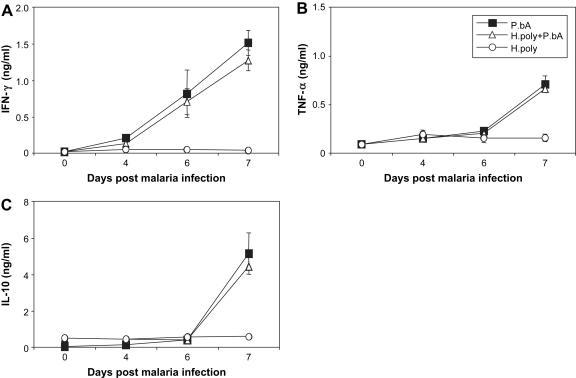
Concurrent *H. polygyrus* infection does not alter the systemic inflammatory response. Groups of C57BL/6 mice were inoculated as in Fig. 1 and levels of circulating IFN-γ (A), TNF-α (B) and IL-10 (C) were measured in sera by sandwich ELISA. Mean and SEM are shown.

As the release of pro-inflammatory cytokines is believed to contribute to the induction of CM, we quantified the levels of IFN-γ, TNF-α and IL-10 in sera. The data in Fig. 2C show that helminth infected mice had significantly higher levels of circulating IL-10 at the time of the initiation of malaria infection compared to mice without helminth infection (*H. polygyrus* infected: 0.508 ± 0.077 ng/ml; uninfected: 0.047 ± 0.003 ng/ml, *p* < 0.005). This fact did not, however, alter the increase of malaria-induced IFN-γ and TNF-α as the malaria infection progressed in co-infected mice. At the time of first CM symptoms (day 7) both groups of malaria infected mice showed significantly increased levels of all three cytokines in the circulation as compared to mice with only helminth infection (Fig. 2). These data suggest that although this helminth infection cause increased systemic IL-10 levels, it does not induce sufficient systemic Th2 or T regulatory activity to alter the cytokine response induced by malaria infection.

### 3.3 Concurrent *H. polygyrus* infection does not induce cerebral malaria in CM-resistant BALB/c mice

**Fig. 3:**
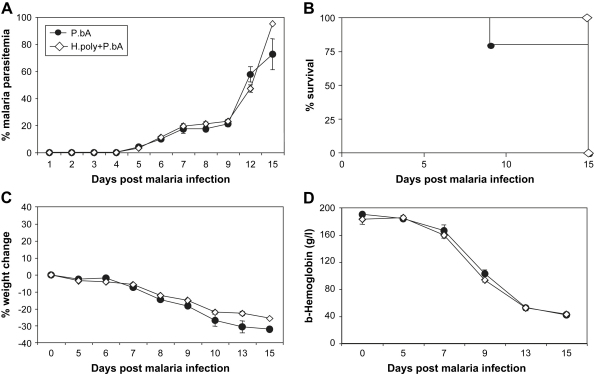
Concurrent *H. polygyrus* infection does not induce cerebral malaria in resistant BALB/c mice. Groups of BALB/c mice were inoculated as in Fig. 1. The development of malaria parasitemia (A), mortality (B), changes in body weight (C) and hemoglobin levels (D) were assessed. Mean and SEM are shown.

In contrast to C57BL/6 mice, BALB/c mice are resistant to cerebral malaria [2] and instead succumb to severe anemia at later stages of PbA infection. In order to examine if a concurrent *H. polygyrus* infection would induce cerebral malaria in a CM-resistant strain of mice, we infected BALB/c mice using the same protocol as above. Again, there was no difference between malaria only and *H. polygyrus* co-infected animals. None of the mice developed cerebral symptoms but all mice from both groups succumbed to severe anemia and high parasitemia around 15 days post infection (Fig. 3).

In conclusion, our data show that we are unable to provide evidence that a chronic gastro-intestinal helminth infection such as *H. polygyrus* can exacerbate, or protect against, CM in a mouse model.

## 4 Discussion

The possible interaction between helminths and malaria has been the subject of a number of animal and human studies in recent years. Animal models have demonstrated significant effects of co-infections between various types of helminths and murine malarias, in most cases reporting exacerbation of malarial disease while some studies report amelioration (reviewed in Ref. [10]). Human studies have largely mirrored the sometimes contradictory results reported from animal models, with some studies reporting that children with helminth infections have increased risk of clinical malaria or increased risk of severe malaria, while other studies have reported lower *P. falciparum* densities or less clinical disease (reviewed in Refs. [10] and [11]). Such range of contrasting results in human cohorts are likely to be due to, at least in part, the fact that all these studies differ somewhat in cohort definition, experimental design, definition of clinical episodes and severity, as well as in area-specific differences of transmission intensity of both helminths and malaria. With respect to cerebral malaria, studies from Thailand have reported dose-dependent protection against cerebral malaria by helminth infections, in particular *Ascaris lumbricoides* [14–16]. Interestingly, the same group reported an increased likelihood of *P. falciparum* infection in helminth co-infected patients [17], suggesting that in Thai adults, infection with intestinal helminths is associated with an increased risk of non-severe malaria but with protection against cerebral disease.

Our data using a mouse model of chronic intestinal nematode infection, *H. polygyrus* and the murine CM model of *P. berghei* ANKA revealed no interaction between the two infections. Susceptible C57BL/6 mice carrying a helminth infection were not protected against CM, nor did they develop an altered course of parasitemia. Furthermore, co-infection of CM-resistant BALB/c mice did not result in the development of CM or any alteration of the course of malaria in these animals. *H. polygyrus* is a strong inducer of Th2 and T regulatory cells, not only in the intestine but also at peripheral sites such as lungs and spleen ([7–9] and our unpublished observations). We chose to initiate the *P. berghei* infection 14 days after *H. polygyrus* infection to allow for the establishment of the helminth-induced T cell response. Interestingly, previous studies have demonstrated a protective role for IL-10 in CM [18,19], providing the possibility that helminth-induced IL-10 production may have a beneficial effect on murine CM. Despite this, the established helminth response did not affect the outcome of infection, or the systemic cytokine production induced by the malaria infection. As such, we cannot confirm the protective effects of helminths reported in human CM using our murine model. However, it may be that additional factors, such as nutrition and exposure to other pathogens might be involved in the protective effects observed in the Thai cohorts.

CD4^+^ CD25^+^ regulatory T cells have recently been described as playing a detrimental role in human [3], and murine malaria infection [4]. Importantly, depletion of Tregs during PbA infection prevented the development of CM [4]. Given the fact that *H. polygyrus* infection is a strong inducer of T regulatory cells [7–9] it was feasible to hypothesize that a concurrent *H. polygyrus* infection would exacerbate the course of PbA infection in the mouse. However, the lack of effect observed in concurrent *H. polygyrus* and PbA infection suggest that the induction of T regulatory cells by another pathogen is not sufficient to exacerbate, or ameliorate, CM in the mouse. It is presently not clear whether the T regulatory cells observed during malaria infection are antigen specific, similar to those observed during *Leishmania* infection [20], or simply natural occurring Tregs predisposed to self-antigen recognition. It may be the case that the lack of effect of *H. polygyrus*-induced Tregs on PbA CM is reflecting a need for antigen specificity in the pathogenic malaria response, or it may be a case of tissue localization.

In conclusion, we have been unable to identify a major *in vivo* role for a chronic gastro-intestinal helminth infection, such as *H. polygyrus*, in the development of experimental cerebral malaria. However, based on these experiments using one species of helminth we cannot exclude the possibility that infections with other species of helminths, or combinations thereof, may still have a role to play in the pathogenesis of malaria. Research using animal models of parasitic infections will no doubt continue to provide valuable information concerning protection and pathology associated with the induction of the potent T helper and T regulatory responses to parasitic infections, information that can be utilised and taken forward to population studies in the field.
